# The threshold value for identifying insulin resistance (HOMA-IR) in an admixed adolescent population: A hyperglycemic clamp validated study

**DOI:** 10.20945/2359-3997000000533

**Published:** 2022-12-01

**Authors:** Cleliani de Cassia da Silva, Mariana Porto Zambon, Ana Carolina Junqueira Vasques, Daniella Fernandes Camilo, Maria Ângela Reis de Góes Monteiro Antonio, Bruno Geloneze

**Affiliations:** 1 Universidade Estadual de Campinas Laboratório de Investigação em Metabolismo e Diabetes Centro de Diagnóstico e Pesquisa Gastroenterológica Campinas SP Brasil Laboratório de Investigação em Metabolismo e Diabetes (Limed), Centro de Diagnóstico e Pesquisa Gastroenterológica (Gastrocentro), Universidade Estadual de Campinas (Unicamp), Campinas, SP, Brasil; 2 Universidade Estadual de Campinas Programa de Pós-graduação em Saúde da Criança e do Adolescente Faculdade de Ciências Médicas Campinas SP Brasil Programa de Pós-graduação em Saúde da Criança e do Adolescente, Faculdade de Ciências Médicas, Universidade Estadual de Campinas (Unicamp), Campinas, SP, Brasil; 3 Universidade Estadual de Campinas Departamento de Pediatria Campinas SP Brasil Departamento de Pediatria, Universidade Estadual de Campinas (Unicamp), Campinas, SP, Brasil; 4 Universidade Estadual de Campinas Faculdade de Ciências Aplicadas Limeira SP Brasil Faculdade de Ciências Aplicadas, Universidade Estadual de Campinas (Unicamp), Limeira, SP, Brasil; 5 Instituto Nacional de Ciência e Tecnologia da Obesidade e Diabetes Campinas SP Brasil Instituto Nacional de Ciência e Tecnologia da Obesidade e Diabetes, Campinas, SP, Brasil

**Keywords:** Homeostasis model assessment, insulin resistance, glucose clamp technique, adolescents

## Abstract

**Objectives:**

To validate the homeostasis model assessment (HOMA) of insulin resistance (IR) as a surrogate to the hyperglycemic clamp to measure IR in both pubertal and postpubertal adolescents, and determine the HOMA-IR cutoff values for detecting IR in both pubertal stages.

**Subjects and methods:**

The study sample comprised 80 adolescents of both sexes (aged 10-18 years; 37 pubertal), in which IR was assessed with the HOMA-IR and the hyperglycemic clamp.

**Results:**

In the multivariable linear regression analysis, adjusted for sex, age, and waist circumference, the HOMA-IR was independently and negatively associated with the clamp-derived insulin sensitivity index in both pubertal (unstandardized coefficient – B = −0.087, 95% confidence interval [CI] = −0.135 to −0.040) and postpubertal (B = −0.101, 95% CI, −0.145 to −0.058) adolescents. Bland-Altman plots showed agreement between the predicted insulin sensitivity index and measured clamp-derived insulin sensitivity index in both pubertal stages (mean = −0.00 for pubertal and postpubertal); all *P* > 0.05. The HOMA-IR showed a good discriminatory power for detecting IR with an area under the receiver operator characteristic curve of 0.870 (95% CI, 0.718-0.957) in pubertal and 0.861 (95% CI, 0.721-0.947) in postpubertal adolescents; all *P* < 0.001. The optimal cutoff values of the HOMA-IR for detecting IR were > 3.22 (sensitivity, 85.7; 95% CI, 57.2-98.2; specificity, 82.6; 95% CI, 61.2-95.0) for pubertal and > 2.91 (sensitivity, 63.6; 95% CI, 30.8-89.1, specificity, 93.7; 95%CI, 79.2-99.2) for postpubertal adolescents.

**Conclusion:**

The threshold value of the HOMA-IR for identifying insulin resistance was > 3.22 for pubertal and > 2.91 for postpubertal adolescents.

## INTRODUCTION

The increase in the prevalence of type 2 diabetes mellitus (1,2) and metabolic syndrome (3) in children and adolescents is an important public health concern. Insulin resistance has a key role in the pathogenesis of type 2 diabetes mellitus (1,4) and metabolic syndrome (3,5), which can lead to the development of coronary artery disease (3,6). Hence, a valid, practical, and accessible method of assessing insulin resistance in this age group must be developed to monitor its progression over time, to identify adolescents at risk of developing associated factors, and to establish strategies for preventing and mitigating the transition from normal glucose tolerance to impaired fasting glucose and type 2 diabetes mellitus.

Insulin resistance can be assessed *in vivo* by several methods. The euglycemic-hyperinsulinemic clamp technique is considered the gold standard for assessing insulin sensitivity/resistance (7,8). However, it is not applicable to large-scale epidemiological studies or clinical practice due to being a complex, invasive, expensive, and time-consuming method (8).

The homeostasis model assessment (HOMA) of insulin resistance (IR) is a surrogate marker that estimates insulin resistance based on basal measurements of plasma insulin and glucose (9). It has been widely validated and used in clinical and epidemiological studies of adult populations (10–12). The HOMA-IR has also been validated as a surrogate to the clamp technique as a measure of insulin resistance in adolescents (13–17). However, to our knowledge, studies have not separately validated the HOMA-IR in pubertal and postpubertal adolescents. Cross-sectional and longitudinal studies have shown that a significant physiological change in insulin sensitivity occurs during the transition from late childhood throughout adolescence, with increased insulin resistance at the onset of puberty and subsequent normalization towards the end of pubertal development (18,19).

This study aimed to validate the HOMA-IR as a surrogate to the hyperglycemic clamp technique to measure insulin resistance in both pubertal and postpubertal adolescents; and determine the HOMA-IR cutoff values for detecting insulin resistance in both pubertal stages.

## SUBJECTS AND METHODS

### Study design and participants

This study used data from the Brazilian Metabolic Syndrome Study (BRAMS), a cross-sectional study conducted in the state of São Paulo, Brazil. The BRAMS studied the insulin resistance in an intentional non-probabilistic sample composed of adolescents aged from 10 to 19 years and 11 months. Out of 1,033 enrolled participants in the BRAMS study, data from 80 adolescents (aged 10-18 years, 40 females) who underwent the hyperglycemic clamp technique were analyzed. The adolescents were recruited from public schools and the University of Campinas Teaching Hospital. The following exclusion criteria were applied: prepubertal children due to the small sample size, pregnancy, use of either systemic corticosteroids or drugs with hypoglycemic properties, malnutrition, hepatopathy, nephropathy, metabolic disorders (e.g., hypothyroidism, hyperthyroidism, and type 1 and 2 diabetes), genetic syndrome diagnosis, and delayed neuropsychomotor development.

The study was approved by the Research Ethics Committee of the School of Medical Sciences of the University of Campinas (protocol number 900/2010, CAAE: 0696.0.146.146-10) and was performed following the ethical principles of the Declaration of Helsinki 1961 (revised in 2008). All participants’ legal guardians signed an informed consent form.

### Clinical evaluation

Pubertal development was assessed by self-assessments (20) according to Tanner's criteria (21). The self-assessment method in the BRAMS study has been reported in detail elsewhere (22). Participants were divided into two groups: pubertal (Tanner II-IV) and postpubertal (Tanner V).

### Anthropometric and body composition assessments

The body mass index (BMI) was calculated as weight (kilograms) divided by height in meters squared. The BMI-for-age *z-*score was calculated using the Epi Info version 3.5.2 software (Centers for Disease Control and Prevention, Atlanta, Georgia, USA). The nutritional status was defined using the Centers for Disease Control and Prevention criteria (23). Waist circumference was measured at the midpoint between the lower margin of the last palpable rib and the top of the iliac crest (24). The amount of lean body mass was determined using tetrapolar bioimpedance (Biodynamics, model 310, Shoreline, Washington, USA) (25).

### Biochemistry assessment

Blood samples were collected after a 12-hour overnight fast. Plasma glucose was measured by using enzymatic colorimetric method (monoreagent K082; Bioclin Systems II^®^, Quisaba, Bioclin, Belo Horizonte, MG, Brazil). Insulin levels were analyzed by using a human insulin enzyme-linked immunosorbent assay kit (EZHI-14K; Millipore; St. Louis, Missouri, USA).

### Insulin resistance assessment

Participants underwent a 2-hour hyperglycemic clamp (with blood glucose acutely raised and maintained at approximately 225 mg/dL; to convert to millimoles per liter, multiply by 0.0555) according to the protocol previously described by Arslanian (8). The insulin sensitivity index (ISI) from the hyperglycemic clamp technique was calculated as the mean exogenous glucose infusion rate from 60 to 120 minutes of the clamp technique minus the urinary glucose excretion, divided by the mean insulin concentration of five determinations during the same time period, and it was then corrected for lean body mass (LBM) (ISI_LBM_; milligrams of glucose infused per kilogram of lean body mass per minute, multiplied by 100) (8,26).

The HOMA-IR index was calculated as the product of the fasting plasma insulin level (in milliunits per liter) and the fasting plasma glucose level (in millimoles per liter), divided by 22.5 (9).

### Statistical analysis

The Shapiro-Wilk test was used to check the distribution of variables. Data are reported as the mean ± standard deviation or median (interquartile range). Relationship between two variables was evaluated with the Spearman's correlation coefficient. We used multivariable linear regression models to evaluate the associations between the HOMA-IR and the clamp-derived ISI (independent variables, sex, age, and HOMA-IR in Model 1 and the variables in Model 1 plus waist circumference in Model 2). Residuals were evaluated for normal distribution by the Shapiro-Wilk test. Preliminary prediction models demonstrated non-normality of the residuals and clamp-derived ISI was therefore transformed to the logarithmic scale. The agreement between the predicted ISI and measured clamp-derived ISI_LBM_ was evaluated by Bland-Altman plots (27). For each plot, average bias and 95% limits of agreement were estimated, with results analyzed further by a One-sample T-test to assess the significance of any bias between measured clamp-derived ISI_LBM_ and predicted ISI (28). The bias represents the mean difference between the two methods (29). For a method to be considered of good agreement, the mean differences should not different from zero (29). The area under the receiver-operating characteristic (ROC) curve (AUC), sensitivity, specificity, positive predictive value, and negative predictive value were calculated to evaluate the accuracy of the HOMA-IR for detecting insulin resistance in both pubertal and postpubertal adolescents. The optimal cutoff points of the HOMA-IR were obtained from the Youden index, which is defined as the maximum sensitivity + specificity − 1 (30). To classify insulin resistance for the ROC curve analysis, the cutoff of the insulin sensitivity index corrected for lean body mass (lower 10th percentile) derived from the normal-weight group was used (cutoff, < 0.08 mg/kg_LBM_/min per mU/L for the pubertal and < 0.07 mg/kg_LBM_/min per mU/L for the postpubertal adolescents). Statistical analyses were performed using IBM SPSS Statistics for Windows, version 20.0 (IBM Corporation, Armonk, NY, USA) and MedCalc for Windows, version 18.5 (MedCalc Software, Ostend, Belgium). In all statistical tests, *P* values < 0.05 were considered significant.

## RESULTS

Table 1 shows clinical, anthropometric, and biochemical data.

**Table 1:** Characteristics of the study population

Characteristic		Pubertal (n = 37)	Postpubertal (n = 43)
Age, years		13.3 ± 1.8	15.9 ± 2.1
Sex, %			
	Female	37.8	60.5
	Male	62.2	39.5
Nutritional status, %			
	Normal weight	27.0	39.5
	Overweight	8.1	14.0
	Obesity	64.9	46.5
Body mass index, kg/m^2^		27.9 (21.7-31.0)	27.6 (21.8-32.2)
Body mass index-for-age z-score		1.92 (0.84-2.27)	1.57 (0.56-2.06)
Waist circumference, cm		92.5 (73.5-104.0)	90.0 (73.5-97.0)
Lean body mass, kg		48.7 ± 12.7	52.4 ± 14.0
Plasma glucose, mg/dL		90 ± 7	86 ± 7
Plasma insulin, mU/L		13.4 (8.0-19.6)	10.3 (6.9-12.7)
HOMA-IR		2.81 (1.67-4.32)	2.11 (1.49-2.83)
Insulin sensitivity index, mg/kg_LBM_/min per mU/L × 100		0.10 (0.06-0.29)	0.13 (0.06-0.24)

HOMA-IR: homeostasis model assessment of insulin resistance; LBM: lean body mass.

SI conversion factors: to convert glucose to millimoles per liter, multiply by 0.0555; and to convert insulin to picomoles per liter, multiply by 6.

Data are presented as the mean ± standard deviation or as the median (interquartile ranges).

### Relationship of the HOMA-IR index and clamp-derived insulin sensitivity index

The HOMA-IR index showed a strong correlation with the clamp-derived ISI in both the pubertal (r = −0.77; *P* < 0.001) and postpubertal (r = −0.83; *P* < 0.001) adolescents.

### Association between the clamp-derived insulin sensitivity index and HOMA-IR index

In the multivariable linear regression analysis, adjusted for sex and age, the HOMA-IR index was independently and negatively associated with the clamp-derived ISI in both the pubertal and postpubertal adolescents (Table 2). The HOMA-IR index remained negatively associated with the clamp-derived ISI, in both the pubertal and postpubertal adolescents, even after further adjustment for waist circumference (Table 2).

**Table 2:** Association between the HOMA-IR index and clamp-derived insulin sensitivity index in the pubertal and postpubertal adolescents – multivariable linear regression analysis

Dependent Variables	Pubertal						
	Model	Independent variables	B (SE)	95% CI	β	*P*-value	R^2^ for model
ISI, mg/kg_LBM_/min per mU/L × 100^a^	1	Constant	−0.355 (0.467)	−1.304 to 0.594	-	0.452	0.539
		HOMA-IR	−0.140 (0.024)	−0.188 to −0.092	−0.725	<0.001	0.701
		Sex^b^	0.056 (0.122)	−0.192 to 0.304	0.056	0.650	
		Age, years	−0.011 (0.033)	−0.78 to 0.055	−0.042	0.728	
	2	Constant	0.501 (0.434)	−0.383 to 1.385	-	0.257	
		HOMA-IR	−0.087 (0.023)	−0.135 to −0.040	−0.451	0.001	
		Sex^b^	0.206 (0.106)	−0.010 to 0.422	0.206	0.061	
		Age, years	−0.005 (0.027)	−0.060 to 0.049	−0.019	0.847	
		Waist circumference, cm	−0.013 (0.003)	−0.020 to −0.007	−0.489	<0.001	

Dependent Variables	Postpubertal						
	Model	Independent variables	B (SE)	95% CI	β	*P*-value	R^2^ for model
ISI, mg/kg_LBM_/min per mU/L × 100^a^	1	Constant	−0.173 (0.334)	−0.849 to 0.503	-	0.607	0.609
		HOMA-IR	−0.148 (0.020)	−0.188 to −0.109	−0.763	<0.001	0.709
		Sex^b^	0.064 (0.091)	−0.120 to 0.248	0.073	0.488	
		Age, years	−0.024 (0.021)	−0.067 to 0.019	−0.117	0.270	
	2	Constant	0.495 (0.346)	−0.205 to 1.195	-	0.160	
		HOMA-IR	−0.101 (0.021)	−0.145 to −0.058	−0.522	<0.001	
		Sex^b^	0.114 (0.081)	−0.050 to 0.278	0.130	0.166	
		Age, years	−0.025 (0.019)	−0.063 to 0.013	−0.121	0.193	
		Waist circumference, cm	−0.009 (0.002)	−0.014 to −0.004	−0.402	0.001	

B: unstandardized coefficient; SE: standard error; β: standardized coefficient; CI: confidence interval; HOMA-IR: homeostasis model assessment of insulin resistance; ISI: insulin sensitivity index; LBM: lean body mass.

a Logarithmic transformation was performed.

b 0 represents the female and 1 the male.

### Agreement between the predicted ISI and measured clamp-derived ISI_LBM_

**Figure 1:**
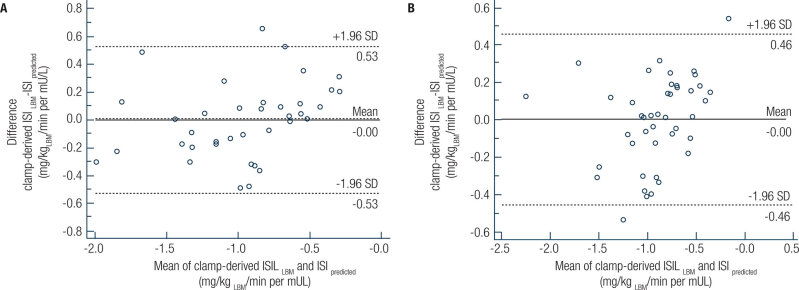
Bland-Altman plots showing the agreement between the predicted insulin sensitivity index and measured clamp-derived insulin sensitivity index in pubertal (**A**) and postpubertal (**B**) adolescents.

The Bland-Altman plot shows a bias equal to zero (Figure 1). The mean differences between predicted ISI and measured clamp-derived ISI_LBM_ were not significantly different from zero in both pubertal (t(36) = 0.00, *P* > 0.05) and postpubertal (t(42) = 0.00, *P* > 0.05) adolescents.

### Accuracy and Cutoff point of the HOMA-IR index for detecting insulin resistance

**Figure 2:**
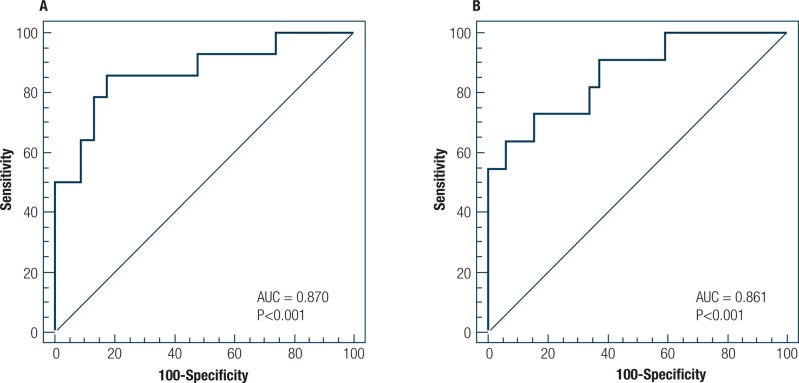
Receiver operating characteristic curves of discriminative ability to detect insulin resistance in pubertal (**A**) and postpubertal (**B**) adolescents.

The analysis of the ROC curves showed that the HOMA-IR index had a good discriminatory power for detecting insulin resistance in the pubertal (AUC ± standard error [SE], 0.870 ± 0.06; 95% confidence interval [CI], 0.718-0.957; *P* < 0.001) and postpubertal adolescents (AUC ± SE, 0.861 ± 0.06; 95% CI, 0.721-0.947; *P* < 0.001) (Figure 2). The optimal cutoff values of the HOMA-IR index for detecting insulin resistance were > 3.22 (sensitivity, 85.7 [95% CI, 57.2-98.2]; specificity, 82.6 [95%CI, 61.2-95.0]; positive predictive value (+PV), 75.0 [95% CI, 54.6-88.2]; negative predictive value (−PV), 90.5 [95% CI, 72.2-97.2]; Youden's index, 0.68) for the pubertal and > 2.91 (sensitivity, 63.6 [95% CI, 30.8-89.1], specificity, 93.7 [95% CI, 79.2-99.2]; +PV, 77.8 [95% CI, 46.0-93.5]; −PV, 88.2 [95% CI, 77.3-94.3]; Youden's index, 0.57) for the postpubertal adolescents.

## DISCUSSION

This study of pubertal and postpubertal adolescents indicates that the HOMA-IR index is strongly related with the clamp-derived ISI in both pubertal stages. To our knowledge, this is the first study to explore the association between the HOMA-IR index and the clamp-derived ISI in these two pubertal stages separately. We found that the HOMA-IR index was negatively associated with clamp-derived insulin sensitivity, even after adjustment for waist circumference, in both pubertal and postpubertal adolescents. Additionally, Bland-Altman plots showed agreement between the predicted ISI and measured clamp-derived ISI_LBM_ in both pubertal stages. Finally, we found that the HOMA-IR index was capable of accurately detecting insulin resistance in both pubertal and postpubertal adolescents. The cutoff points for detecting insulin resistance using the HOMA model were different between the pubertal (>3.22) and postpubertal (>2.91) adolescents.

Studies have compared the HOMA-IR index with clamp-derived measures in pediatric populations (13–17), and as well as in this study, they found a significant correlation between the two methods. However, previous studies have not demonstrated whether the HOMA-IR is capable of separately estimating insulin resistance in pubertal and postpubertal adolescents. Although Gungor and cols. (14) reported correlations for pubertal adolescents, they defined pubertal adolescents as Tanner stages II to V. However, in our study, adolescents were considered as pubertal if they presented Tanner stages II-IV and postpubertal if they presented Tanner stage V. We divided the adolescents into these two development stages based on the study by Moran and cols. (18), who demonstrated that insulin resistance measured by the glucose clamp technique increases significantly at Tanner stages II, III, and IV but decreases to near prepubertal (Tanner stage I) levels at Tanner stage V.

The multivariable linear regression analysis showed that the results of the HOMA-IR were independently and negatively associated with insulin sensitivity results of the clamp technique in the pubertal and postpubertal adolescents. These results indicate the validity of the HOMA-IR in explaining the insulin resistance results of the hyperglycemic clamp technique in both pubertal stages. Additionally, the analysis of the ROC curve revealed that the HOMA-IR index could accurately detecting insulin resistance in both pubertal and postpubertal adolescents.

The cutoff identified for pubertal adolescents in our study (>3.22) was similar to previously reported values (3.16 to 3.3) (31,32), whereas the cutoff for postpubertal adolescents (>2.91) was higher than previously reported value (2.7) (32). These differences may be related to BMI differences, population age, although they are primarily related to the accuracy of the methodology used for determining the cutoff point (hyperglycemic clamp versus oral glucose tolerance test (31) or 95th percentile of the HOMA-IR (32)).

A potential limitation of the current study is its cross-sectional design, which does not allow for inferences of causality. Another limitation is the use of the hyperglycemic clamp technique to evaluate insulin resistance. Although the hyperglycemic clamp technique is not the gold standard for estimating insulin resistance, studies comparing this technique with the euglycemic-hyperinsulinemic clamp technique (gold standard for quantifying insulin resistance) reported an excellent correlation between both clamp techniques in children and adolescents (14,26,33). The evaluation of sexual maturation was performed via self-assessments (20,34) to increase the participation rate, due to privacy concerns, cultural, and emotional factors. Studies that evaluated the agreement between self-assessment sexual maturation and physical examination performed by a physician (20,34) suggest that the self-assessment can be used in epidemiologic studies for evaluating sexual maturation when the physician exam is impossible. Also, this study did not report race/ethnicity-stratified results, because Brazil has one of the most admixed populations and this distinction is unfeasible.

In summary, the threshold value of the HOMA-IR for identifying insulin resistance was > 3.22 for pubertal and > 2.91 for postpubertal adolescents. The HOMA-IR is a low-cost approach with potential clinical and epidemiological applications and these cutoff points can improve the detection and control of metabolic diseases in pubertal and postpubertal adolescents.
